# Improving and Sustaining Resident Physician Handover

**DOI:** 10.7759/cureus.53413

**Published:** 2024-02-01

**Authors:** Devin Reilly, Shaefali Shandilya, Blair Streater, Bettina Aprile, Justen M Aprile

**Affiliations:** 1 Pediatrics, Penn State Health Children's Hospital, Hershey, USA; 2 Family and Community Medicine, Penn State Health Milton S. Hershey Medical Center, Hershey, USA

**Keywords:** undergraduate and graduate medical education, safety patient, quality improvement research, health communication, medical resident education

## Abstract

Background

Handoffs serve a critical patient safety function in the transition between caregivers. In 2006, the Joint Commission on Accreditation of Healthcare Organizations strongly recommended the implementation of “a standardized approach to ‘handoff’ communications, including an opportunity to ask and respond to questions.” Numerous studies have investigated the quality and efficacy of patient handoffs and the utility of structured handoff curriculums, particularly in the context of patient safety and outcomes.

Objective

The pediatric residents at Penn State Health (PSH) did not utilize a formal written or verbal handoff tool. Our study facilitated the design of a comprehensive handoff curriculum, including verbal and written components, and the implementation of faculty and multidisciplinary care team involvement coupled with resident training and observations. We investigate the impact of this curriculum longitudinally utilizing validated tools completed by external observers as well as the residents themselves.

Methods

Prior to SAFETIPS being implemented, residents at a mid-sized Pediatric program were observed giving handovers at various intervals to understand baseline habits. Residents were then educated with the SAFETIPS curriculum and again observed. Trained observers of the handover process completed a validated evaluation form concentrating on seven key domains necessary for effective handover and communication; residents involved in the handover also completed a validated evaluation form. Consent for the project was implied with the observer's presence during the process and our study was exempt from full IRB consideration given its quality improvement design. A mix of summary statistics, stacked dot plots, mixed effects regression, and joint F tests were used to analyze data.

Results

Mean values on all sections of the handover evaluation Likert scale completed by trained observers tended to increase over time; the variance in responses was likewise much smaller at later time periods. Similarly, all sections of the evaluation tools completed by the resident physicians themselves showed significantly increased scores from pre- to post-implementation of our curriculum. Data revealed a plateauing of results toward later time points suggestive of skills mastery and sustained improvements.

Conclusion

Our findings suggest that the introduction of a structured handoff curriculum correlated with improved communication among residents, and such improvements were sustained over time.

## Introduction

The transfer of patient information between health care providers, or physician handover, is a critically important area of medicine. “Sign-out,” “hand-off,” or “handover” is defined as “the transfer of professional responsibility and accountability for some or all aspects of care for a patient, or group of patients, to another person or professional group on a temporary or permanent basis" [1-12]. This practice has become increasingly relevant given the focus of the Accreditation Council for Graduate Medical Education (ACGME) on reducing resident duty hours. The reduction in duty hours has led to an increased number of resident shifts and thus an increased frequency of handovers, which creates vulnerabilities in patient safety due to insufficient, incomplete, or inaccurate communication.

Since the implementation of reduced duty hours, many faculty members and program directors within clinical learning environments (CLEs) have voiced concerns regarding patient safety vulnerabilities prompted by institutional efforts to comply with new duty hour requirements. Even before the new duty hours, it was reported that as many as 100,000 people die each year because of injuries secondary to erroneous medical care, the majority of which are related to physicians’ communication breakdown [12]. It has been estimated that nearly 80% of serious medical errors stem from miscommunication during patient transfers of care [7]. Approximately 4,000 handoffs occur per day, for a total of 1.6 million per year, which makes high-fidelity hand-offs critical to improving patient safety [8]. This data coupled with the increased vulnerability in patient safety caused by more frequent handovers makes standardizing the information contained in handovers, as well as the handover process itself essential.

Among many publications that acknowledge this is the 2016 ACGME Clinical Learning Environment Review (CLER). The CLER's aims are to improve the clinical setting within which learners are immersed, improve the quality of resident education, and educate residents on how to become lifelong learners as it relates to patient care [3,4,13-18]. Further, the ultimate goal of the CLER report is to improve patient care while optimizing the educational experience for resident and fellow physician learners. In order to achieve this aim, the report has seven focus areas including patient safety, health care quality, health care disparities, supervision, fatigue management and duty hours, professionalism, and care transitions. Per the CLE's review, in regard to patient safety and care transitions, most CLEs did not have a standardized approach to facilitating resident and fellow hand-offs at change of duty that included essential elements of safe, reliable transitions of care.

In an effort to improve patient safety by way of standardized handover strategies, multiple systematic reviews regarding handover systems and mnemonics exist [1-10,19-30]. One clinical process curriculum is called SAFETIPS (“S”tats, “A”ssessment, “F”ocused plan, “E”xam, “T”o do, “I”f/then, “P”ointers/pitfalls, “S”ick-o-meter). The aforementioned is a tool developed and used to help residents recall and provide pertinent information to aid in high-quality, standardized handovers. Studies using SAFETIPS have demonstrated an improvement in the sustained inclusion of essential information and successful identification of at-risk patients, while also providing the necessary information to better anticipate potentially harmful patient events [13,14].

Unique to our study, and underpinning its rationale and significance, is the role of the SAFETIPS curriculum in meeting the goals of the CLER report set forth in 2016. While the CLER report discusses several themes, SAFETIPS, and resident handover addresses and meets the CLER goals in the following three areas: 1) Engage learners (i.e., residents) in system-based improvements, 2) Engage learners in patient safety, 3) Engage learners across the health care discipline (i.e., resident and nursing teams) [4,15-18]. In addition to the implementation of the SAFETIPS handover bundle, we aimed to not only standardize the written and verbal handover process itself but also study the impact of that standardization over time through the use of two validated tools looking at seven key domains necessary for effective handover and communication [23,24].

This study expands on our previous work in which one of our authors explored improved resident communications at another institution [10]. Our current work, while again studying pediatric residents, assesses the impact and sustainability of a handoff curriculum in a medium-sized pediatric residency at a tertiary care center.

## Materials and methods

**Figure 1:**
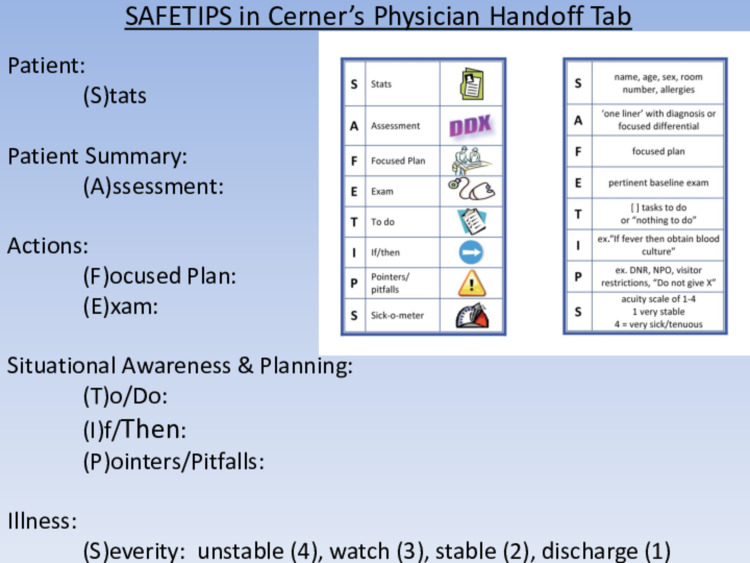
SAFETIPS mnemonic DNR = Do Not Resuscitate, NPO = Nothing By Mouth

A curriculum known as SAFETIPS was introduced to the Penn State Hershey Pediatric Residency (“S”tats, “A”ssessment, “F”ocused plan, “E”xam, “T”o do, “I”f/then, “P”ointers/pitfalls, “S”ick-o-meter). Utilizing SAFETIPS as the backbone of our project, a handover “bundle” was successfully created and implemented with the following components: restructured and standardized time/location for AM and PM handover, SAFETIPS curriculum (verbal and written) implemented and refresher courses were held at the beginning of each academic block (Figure 1), observation of resident handover pre- and post-implementation using a validated handover evaluation Likert scale, and resident evaluation of both written and verbal handover formats utilizing a validated tool.

Study design

The goal of the study was to evaluate resident handover of pediatric patients using an end-of-shift handoff curriculum called SAFETIPS. Penn State is a medium-sized pediatric residency program that utilizes a night float curriculum. Each morning, one night team will hand off to three inpatient teams which include both general and subspecialty service lines. Each evening, that process is reversed. Teams are generally composed of one senior resident and anywhere from one to three interns which may include fourth-year medical students (as acting interns), family medicine interns, and Med/Peds interns.

Prior to SAFETIPS being implemented, residents were observed giving verbal handovers on a number and variety of days and times. It was noted that interns and senior residents frequently signed out separately and at differing times. Residents were then educated with the SAFETIPS curriculum for verbal and written handover, a process that included didactic education sessions as well as simulated practice sessions. After the implementation of SAFETIPS for the verbal handover and implementation of the SAFETIPS format for the written handover, residents were again observed giving verbal handover on a number and variety of days and times. On each day of observation, a trained observer of the verbal handover process completed an evaluation form. Residents also completed an evaluation form at the conclusion of their shift evaluating the quality of the verbal handover as well as an evaluation of the quality of the written handover contained within the EMR.

The study continued over time, resulting in more time periods of observation and evaluation after SAFETIPS was implemented on a rolling basis. The range of dates that form each time period, consisting of four-week blocks, are listed below: pre-implementation: May 2016 to November 2016, post-implementation #1: February 2017 to May 2017, post-implementation #2: September 2017 to November 2017, post-implementation #3: January 2018 to May 2018, post-implementation #4: November 2018.

The trained observer evaluation was recorded on a handover evaluation Likert scale from 0 to 9, with higher scores indicating a more effective, efficient, and complete handover. The validated handover evaluation Likert scale utilized in our project was borrowed, with permission, from a similar study performed in 2013 aimed at the development of a tool to access physician handover [24]. The tool evaluates seven key domains necessary for effective handover and communication: organization/efficiency, communication skills (i.e., understanding confirmed, tasks clear, concrete language), content (i.e., relevant information, clear rationale), clinical judgment (i.e., sick patients identified, anticipatory guidance given), patient-focused (i.e., appropriate patient comments), and setting (i.e., interruptions, distractions). Residents self-evaluated the handoff utilizing the ACGME-created UPDATED evaluation tool [23].

Data

Data was collected through the following means: Medical Student Research (MSR) students (trained in evaluating handover) observing verbal handover and completing a validated handover evaluation Likert scale, residents completing a validated survey regarding the completeness of the written handover system as well as the quality of verbal handover provided to them at the beginning of their overnight shift [18,19].

The data was collected in Redcap separately for residents and observers. Evaluation forms marked as “Practice” or “Test” were excluded. A total of three resident evaluations were excluded because a note indicated that no patients were handed off.

Likert-Scale Handover Evaluations by Observers

The total number of observations for each time period is listed: pre: N=54, post-1: N=33, post-2: N=31, post-3: N=35. No evaluation forms were included for post-implementation time period #4.

UPDATED Evaluations by Residents

The total number of observations for each time period is listed below: pre: N=52, post-1: N=16, post-2: N=23, post-3: N=20, post-4: N=2. The post-implementation time period #4 was excluded from the analysis since it only included 2 observations.

Other Important Notes

To account for times when multiple residents were involved in a patient handover, each resident may provide a separate evaluation form, and the observer may have provided a separate evaluation form for each resident. (Some observers provided only one evaluation form for a given handover session, regardless of the number of residents involved.)

Residents may have been evaluated on different days, within the same time period, and/or between time periods. Residents sometimes acted as observers on days that they were not involved in the handover.

Methods

All analysis was done separately for handover evaluation Likert scales and UPDATED evaluations. Given the limitations in the data set, no attempt was made to link the observations. For the handover evaluation Likert scales, we included the following outcomes: Organization/efficiency score, Communication skills score, Content score, Clinical judgment score, Patient-focused score, Setting score, and Overall quality score.

For the UPDATED tool, the only outcome included was the overall score, which was a summation of seven items on the evaluation form. For each outcome, summary statistics were calculated for each time point. A stacked dot plot was created to show the distribution of handover evaluation Likert scale scores over time. Next to each stacked dot plot, a box plot was also created as another summary of the distribution.

A mixed effects regression model was fit for each outcome. The model contained a fixed effect for the time period (pre, post #1, post #2, and post #3 time periods) and a random effect for observations made on the same date. The model also allowed for different variance estimates for each time period because outcomes tended to exhibit lower variance at later time periods. Note that no attempt was made to account for repeated measurements for the same pediatric resident over time, mainly because the data is not readily available in a format to easily implement this (particularly for the observer data). However, it appears that duplicate residents were only included a small number of times, which the study team believes did not impact results too substantially.

Given the fit of the model, we tested for a significant difference in means across time periods using a joint F-test. If the joint test was significant, then individual follow-up tests were done for each possible time period comparison (6 total tests). For these follow-up tests, we used a Bonferonni correction to identify significant differences. Thus, only tests with p-values < 0.008 were considered significant. The Human Subjects Protection Office determined our project did not meet the definition of human subject research and therefore was exempt from review and approval.

## Results

Resident handover evaluation Likert scales

Table 1 shows the characteristics of each encounter coded in the database stratified by time period and Table 2 shows the summary statistics for each outcome measure stratified by time period.

**Table 1:** Characteristics of each encounter coded in the database stratified by time period. Team A - general pediatric patients. Team B - Hematology/Oncology patients. Team C - a mix of subspecialty patients. Team P - Pulmonary patients.

	Pre (N=54)	Post-1 (N=33)	Post-2 (N=31)	Post-3 (N=35)
Team observed				
No team observed	21 (38.9%)	8 (24.2%)	8 (25.8%)	9 (25.7%)
Team A only	13 (24.1%)	11 (33.3%)	7 (22.6%)	16 (45.7%)
Team B only	7 (13%)	3 (9.1%)	4 (12.9%)	5 (14.3%)
Team C/P only	8 (14.8%)	1 (3%)	8 (25.8%)	5 (14.3%)
Team A and B	1 (1.9%)	0 (0%)	0 (0%)	0 (0%)
Team A and C/P	4 (7.4%)	3 (9.1%)	0 (0%)	0 (0%)
Team A, B, and C/P	0 (0%)	7 (21.2%)	4 (12.9%)	0 (0%)
Did handover start on time?				
No	40 (74.1%)	32 (97%)	22 (71%)	8 (22.9%)
Yes	14 (25.9%)	1 (3%)	9 (29%)	27 (77.1%)
Number of patients handed off				
N	54	33	31	35
Mean (SD)	6.8 (6.73)	11.2 (10.53)	9.2 (13.03)	5.1 (2.91)
Median	4.5	7.0	5.0	4.0
Interquartile range	3.0, 7.0	5.0, 14.0	3.0, 9.0	3.0, 7.0
Range	(1.0-30.0)	(1.0-49.0)	(1.0-47.0)	(2.0-11.0)
Did team utilize a written/verbal handover tool?				
No	52 (96.3%)	0 (0%)	0 (0%)	0 (0%)
Yes	2 (3.7%)	33 (100%)	31 (100%)	35 (100%)
Were any interruptions observed during the handoff process?				
No	13 (24.1%)	4 (12.1%)	7 (22.6%)	16 (45.7%)
Yes	41 (75.9%)	29 (87.9%)	24 (77.4%)	19 (54.3%)

**Table 2:** Summary statistics for each outcome measure stratified by time period.

	Pre, (N=54)	Post-1, (N=33)	Post-2, (N=31)	Post-3, (N=35)
Organization/efficiency score				
Mean (SD)	5.1 (1.27)	7.3 (0.94)	8.1 (1.06)	9 (00)
Median	5	7	8	9
Range	(3-8)	(5-9)	(6-9)	(9-9)
Communication skills score				
Mean (SD)	5.2 (1.53)	6.8 (1.29)	7.7 (1.05)	8.2 (0.51)
Median	5	7	8	8
Range	(2-8)	(4-9)	(5-9)	(7-9)
Content score				
Mean (SD)	5.0 (1.51)	7.0 (1.36)	7.8 (1.45)	8.7 (0.46)
Median	5	7	8	9
Range	(2-8)	(4-9)	(4-9)	(8-9)
Clinical judgment score				
Mean (SD)	4.8 (1.69)	7.2 (1.06)	8.1 (1.45)	9.0 (0.17)
Median	4	7	9	9
Range	(2-9)	(4-9)	(4-9)	(8-9)
Patient focused score				
Mean (SD)	4.9 (1.55)	7.5 (1.25)	8.5 (1.00)	8.8 (0.47)
Median	5	8	9	9
Range	(1-7)	(5-9)	(5-9)	(7-9)
Setting score				
Mean (SD)	3.9 (1.95)	6.1 (1.97)	7.5 (1.36)	8.4 (0.50)
Median	3	7	8	8
Range	(1-8)	(2-9)	(4-9)	(8-9)
Overall quality score				
Mean (SD)	4.7 (1.20)	7.0 (1.13)	8.0 (1.13)	8.6 (0.50)
Median	5	7	8	9
Range	(2-8)	(5-9)	(5-9)	(8-9)

**Figure 2:**
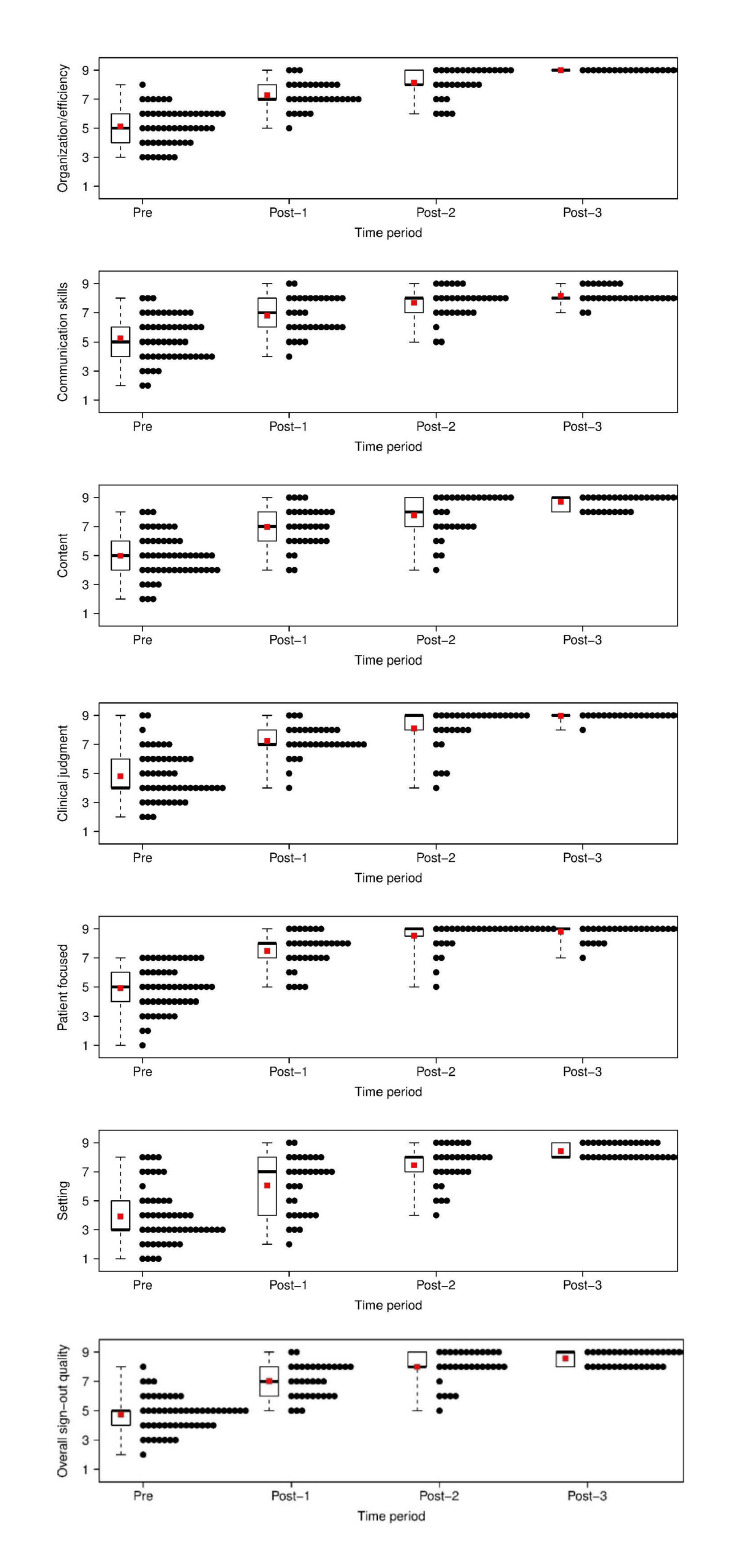
Stacked dot plots (black points) and box plots for each time period for each outcome. The red dot indicates the mean value at each time point. Note that for post-implementation time period 3, some dots are not shown because they trail too far to the right.

These data are also presented graphically using stacked dot plots and box plots (Figure 2).

As demonstrated in Figure 2, the mean value tended to increase over time (possibly plateauing for post-implementation time periods 2 and 3, as expected), and the variance in responses is much smaller at later time periods. In particular, at post-implementation time period 3, most observations were scored at a value of 9.

Organization and efficiency 

The joint F-test was significant (p<0.001), indicating that estimated means differed across time (Table 3).

**Table 3:** Estimated means from fitted model for organization and efficiency.

Parameter	Mean estimate (SE)	95% CI	P-value from joint test
Time period			<0.001
Pre	5.1 (0.18)	(4.8, 5.5)	
Post-1	7.3 (0.16)	(6.9, 7.6)	
Post-2	8.1 (0.19)	(7.7, 8.5)	
Post-3	9.0 (0.01)	(8.9, 9.0)	

Table 4 shows all individual tests by time.

**Table 4:** Follow-up test for each time comparison for organization and efficiency.

Comparison	Difference in means (SE)	95% CI	P-value	Significant by Bonferonni?
Post-1 vs. Pre	2.2 (0.24)	(1.7, 2.6)	<0.001	Yes
Post-2 vs. Pre	3.0 (0.26)	(2.5, 3.5)	<0.001	Yes
Post-3 vs. Pre	3.9 (0.18)	(3.5, 4.2)	<0.001	Yes
Post-2 vs. Post-1	0.9 (0.25)	(0.4, 1.4)	0.001	Yes
Post-3 vs. Post-1	1.7 (0.17)	(1.4, 2.1)	<0.001	Yes
Post-3 vs. Post-2	0.9 (0.19)	(0.51, 1.26)	<0.001	Yes

All comparisons were statistically significant by Bonferonni criteria (p<0.008).

The correction interpretation is, for example, that the organization/efficiency score reported by residents at the post-implementation time period 3 was an average of 3.9 points higher (95% CI: 3.5 to 4.2) than the score at the pre-implementation time period (p<0.001).

Communication skills 

The joint F-test was significant (p<0.001), indicating that estimated means differed across time (Table 5).

**Table 5:** Estimated means from fitted model for communication skills.

Parameter	Mean estimate (SE)	95% CI	P-value from joint test
Time period			<0.001
Pre	5.2 (0.25)	(4.7, 5.7)	
Post-1	6.8 (0.25)	(6.3, 7.3)	
Post-2	7.5 (0.26)	(7.0, 8.0)	
Post-3	8.1 (0.22)	(7.7, 8.6)	

 Table 6 shows all individual tests by time.

**Table 6:** Follow-up tests for each time comparison for communication skills.

Comparison	Difference in means (SE)	95% CI	P-value	Significant by Bonferonni?
Post-1 vs. Pre	1.6 (0.36)	(0.9, 2.3)	<0.001	Yes
Post-2 vs. Pre	2.3 (0.36)	(1.6, 3.1)	<0.001	Yes
Post-3 vs. Pre	3.0 (0.33)	(2.3, 3.6)	<0.001	Yes
Post-2 vs. Post-1	0.7 (0.36)	(0.01, 1.5)	0.047	No
Post-3 vs. Post-1	1.4 (0.33)	(0.7, 2.0)	<0.001	Yes
Post-3 vs. Post-2	0.6 (0.34)	(-0.1, 1.3)	0.07	No

Four comparisons were statistically significant by Bonferonni criteria (p<0.008).

Content

The joint F-test was significant (p<0.001), indicating that estimated means differed across time (Table 7).

**Table 7:** Estimated means from fitted model for content.

Parameter	Mean estimate (SE)	95% CI	P-value from joint test
Time period			<0.001
Pre	4.9 (0.24)	(4.5, 5.4)	
Post-1	7.0 (0.27)	(6.5, 7.5)	
Post-2	7.6 (0.30)	(7.0, 8.2)	
Post-3	8.7 (0.20)	(8.3, 9.2)	

Table 8 shows all individual tests by time.

**Table 8:** Follow-up tests for each time comparison for content.

Comparison	Difference in means (SE)	95% CI	P-value	Significant by Bonferonni?
Post-1 vs. Pre	2.1 (0.36)	(1.4, 2.8)	<0.001	Yes
Post-2 vs. Pre	2.7 (0.38)	(1.9, 3.5)	<0.001	Yes
Post-3 vs. Pre	3.8 (0.31)	(3.2, 4.4)	<0.001	Yes
Post-2 vs. Post-1	0.6 (0.40)	(-0.2, 1.4)	0.12	No
Post-3 vs. Post-1	1.7 (0.34)	(1.1, 2.4)	<0.001	Yes
Post-3 vs. Post-2	1.1 (0.36)	(0.4, 1.8)	0.003	Yes

Five comparisons were statistically significant by the Bonferonni criteria (p<0.008).

Clinical judgment

The joint F-test was significant (p<0.001), indicating that estimated means differed across time (Table 9).

**Table 9:** Estimated means from fitted model for clinical judgment.

Parameter	Mean estimate (SE)	95% CI	P-value from joint test
Time period			<0.001
Pre	4.7 (0.30)	(4.1, 5.3)	
Post-1	7.3 (0.26)	(6.7, 7.8)	
Post-2	7.9 (0.35)	(7.2, 8.5)	
Post-3	8.9 (0.29)	(8.4, 9.5)	

Table 10 shows all individual tests by time.

**Table 10:** Follow-up test for each time comparison for clinical judgment.

Comparison	Difference in means (SE)	95% CI	P-value	Significant by Bonferonni?
Post-1 vs. Pre	2.6 (0.40)	(1.8, 3.4)	<0.001	Yes
Post-2 vs. Pre	3.2 (0.46)	(2.2, 4.1)	<0.001	Yes
Post-3 vs. Pre	4.2 (0.42)	(3.4, 5.1)	<0.001	Yes
Post-2 vs. Post-1	0.6 (0.44)	(-0.3, 1.5)	0.18	No
Post-3 vs. Post-1	1.7 (0.39)	(0.9, 2.4)	<0.001	Yes
Post-3 vs. Post-2	1.1 (0.45)	(0.2, 2.0)	0.020	No

Four comparisons were statistically significant by Bonferonni criteria (p<0.008).

Patient-focused

The joint F-test was significant (p<0.001), indicating that estimated means differed across time (Table 11).

**Table 11:** Estimated means from fitted model for patient focused.

Parameter	Mean estimate (SE)	95% CI	P-value from joint test
Time period			<0.001
Pre	5.0 (0.28)	(4.4, 5.5)	
Post-1	7.6 (0.28)	(7.0, 8.2)	
Post-2	8.4 (0.31)	(7.7, 9.0)	
Post-3	8.9 (0.31)	(8.2, 9.5)	

Table 12 shows all individual tests by time.

**Table 12:** Follow-up test for each time comparison for patient focused.

Comparison	Difference in means (SE)	95% CI	P-value	Significant by Bonferonni?
Post-1 vs. Pre	2.6 (0.39)	(1.8, 3.4)	<0.001	Yes
Post-2 vs. Pre	3.4 (0.42)	(2.6, 4.2)	<0.001	Yes
Post-3 vs. Pre	3.9 (0.42)	(3.1, 4.7)	<0.001	Yes
Post-2 vs. Post-1	0.8 (0.42)	(-0.1, 1.6)	0.08	No
Post-3 vs. Post-1	1.3 (0.42)	(0.4, 2.1)	0.004	Yes
Post-3 vs. Post-2	0.5 (0.44)	(-0.4, 1.4)	0.26	No

Four comparisons were statistically significant by Bonferonni criteria (p<0.008).

Setting

The joint F-test was significant (p<0.001), indicating that estimated means differed across time (Table 13).

**Table 13:** Estimated means for fitted model for setting.

Parameter	Mean estimate (SE)	95% CI	P-value from joint test
Time period			<0.001
Pre	3.7 (0.36)	(3.0, 4.4)	
Post-1	6.4 (0.40)	(5.6, 7.2)	
Post-2	7.2 (0.47)	(6.2, 8.1)	
Post-3	8.3 (0.45)	(7.4, 9.2)	

Table 14 shows all individual tests by time.

**Table 14:** Follow-up test for each time comparison for setting.

Comparison	Difference in means (SE)	95% CI	P-value	Significant by Bonferonni?
Post-1 vs. Pre	2.7 (0.54)	(1.7, 3.8)	<0.001	Yes
Post-2 vs. Pre	3.5 (0.59)	(2.3, 4.7)	<0.001	Yes
Post-3 vs. Pre	4.6 (0.57)	(3.4, 5.7)	<0.001	Yes
Post-2 vs. Post-1	0.8 (0.62)	(-0.5, 2.0)	0.22	No
Post-3 vs. Post-1	1.9 (0.60)	(0.7, 3.1)	0.003	Yes
Post-3 vs. Post-2	1.1 (0.65)	(-0.2, 2.4)	0.09	No

Four comparisons were statistically significant by Bonferonni criteria (p<0.008).

Overall quality

The joint F-test was significant (p<0.001), indicating that estimated means differed across time (Table 15).

**Table 15:** Estimated means for fitted model for overall sign-out quality.

Parameter	Mean estimate (SE)	95% CI	P-value from joint test
Time period			<0.001
Pre	4.7 (0.23)	(4.2, 5.1)	
Post-1	7.1 (0.25)	(6.6, 7.6)	
Post-2	7.7 (0.28)	(7.2, 8.3)	
Post-3	8.6 (0.27)	(8.1, 9.2)	

Table 16 shows all individual tests by time.

**Table 16:** Follow-up test for each time comparison for overall sign-out quality.

Comparison	Difference in means (SE)	95% CI	P-value	Significant by Bonferonni?
Post-1 vs. Pre	2.5 (0.34)	(1.8, 3.2)	<0.001	Yes
Post-2 vs. Pre	3.1 (0.36)	(2.4, 3.8)	<0.001	Yes
Post-3 vs. Pre	4.0 (0.36)	(3.3, 4.7)	<0.001	Yes
Post-2 vs. Post-1	0.6 (0.38)	(-0.1, 1.4)	0.11	No
Post-3 vs. Post-1	1.5 (0.37)	(0.8, 2.2)	<0.001	Yes
Post-3 vs. Post-2	0.9 (0.39)	(0.1, 1.7)	0.027	No

Four comparisons were statistically significant by Bonferonni criteria (p<0.008).

Resident UPDATED evaluations

Table 17 shows the characteristics of each encounter coded in the database stratified by time period and Table 18 shows summary statistics for each component of the evaluation form, including the overall score, stratified by time period.

**Table 17:** Characteristics of each encounter coded in the database stratified by time period. Each column includes the number of evaluations for that time period with the percentage of those total numbers in parenthesis.

	Pre, (N=52)	Post-1, (N=16)	Post-2, (N=23)	Post-3, (N=20)
Team code				
None selected	0 (0%)	2 (12.5%)	1 (4.3%)	3 (15%)
A	17 (32.7%)	5 (31.3%)	6 (26.1%)	4 (20%)
B	14 (26.9%)	3 (18.8%)	5 (21.7%)	4 (20%)
C	15 (28.8%)	4 (25%)	6 (26.1%)	4 (20%)
P	6 (11.5%)	2 (12.5%)	5 (21.7%)	5 (25%)
Day or night team observed				
Missing	13 (.%)	2 (.%)	7 (.%)	11 (.%)
Day	28 (71.8%)	11 (78.6%)	16 (100%)	8 (88.9%)
Night	11 (28.2%)	3 (21.4%)	0 (0%)	1 (11.1%)

**Table 18:** Summary statistics for each component of the evaluation form, including the overall score, stratified by time period.

	Pre, (N=52)	Post-1, (N=16)	Post-2, (N=23)	Post-3, (N=20)
Updated high-risk fields				
0	28 (53.8%)	6 (37.5%)	9 (39.1%)	3 (15%)
1	24 (46.2%)	10 (62.5%)	14 (60.9%)	17 (85%)
Problem list prioritized and updated				
0	18 (34.6%)	3 (18.8%)	1 (4.3%)	0 (0%)
1	22 (42.3%)	5 (31.3%)	5 (21.7%)	6 (30%)
2	12 (23.1%)	8 (50%)	17 (73.9%)	14 (70%)
Diagnosis (presumed) in one-line summary				
0	5 (9.6%)	2 (12.5%)	0 (0%)	0 (0%)
1	47 (90.4%)	14 (87.5%)	23 (100%)	20 (100%)
Anticipated problems clear				
0	10 (19.2%)	2 (12.5%)	0 (0%)	1 (5%)
1	13 (25%)	4 (25%)	4 (17.4%)	6 (30%)
2	29 (55.8%)	10 (62.5%)	19 (82.6%)	13 (65%)
Too much information				
0	18 (34.6%)	3 (18.8%)	0 (0%)	1 (5%)
1	34 (65.4%)	13 (81.3%)	23 (100%)	19 (95%)
Error-free medication list				
0	11 (21.2%)	1 (6.3%)	0 (0%)	1 (5%)
1	41 (78.8%)	15 (93.8%)	23 (100%)	19 (95%)
Directed tasks clear				
0	14 (26.9%)	3 (18.8%)	0 (0%)	1 (5%)
1	38 (73.1%)	13 (81.3%)	23 (100%)	19 (95%)
Overall score				
Mean (SD)	5.8 (1.79)	6.9 (2.50)	8.1 (0.76)	8.0 (1.26)
Median	6	7.5	8	8
Range	(2-9)	(0-9)	(6-9)	(4-9)

**Figure 3:**
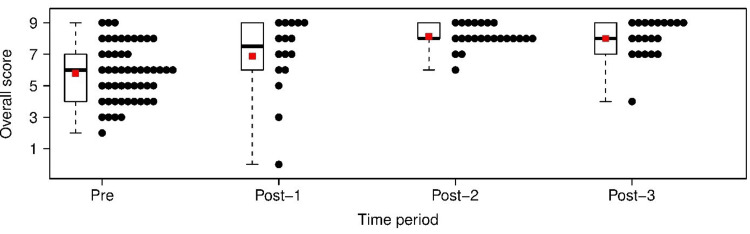
Stacked dot plots (black points) and box plots by time period for overall score. The red dot indicates the mean value at each time point. Note that for post-implementation time period 3, some dots are not shown because they trail too far to the right.

These data are also presented graphically using stacked dot plots and box plots (Figure 3).

Results from the mixed effects model for the overall score are shown in Table 19.

**Table 19:** Estimated mean form fitted model for overall score.

Parameter	Mean estimate (SE)	95% CI	P-value from joint test
Time period			0.002
Pre	5.8 (0.39)	(5.1, 6.6)	
Post-1	6.7 (0.66)	(5.4, 8.0)	
Post-2	8.2 (0.63)	(6.9, 9.4)	
Post-3	8.1 (0.58)	(6.9, 9.3)	

The joint F-test was significant (p=0.002), indicating that estimated means differed across time (Table 20).

**Table 20:** Follow-up test for each time comparison for overall score.

Comparison	Difference in means (SE)	95% CI	P-value	Significant by Bonferonni?
Post-1 vs. Pre	0.9 (0.77)	(-0.7, 2.4)	0.26	No
Post-2 vs. Pre	2.3 (0.74)	(0.9, 3.8)	0.002	Yes
Post-3 vs. Pre	2.3 (0.70)	(0.9, 3.7)	0.002	Yes
Post-2 vs. Post-1	1.5 (0.9)	(-0.4, 3.3)	0.12	No
Post-3 vs. Post-1	1.4 (0.88)	(-0.3, 3.2)	0.11	No
Post-3 vs. Post-2	-0.04 (0.86)	(-1.7, 1.7)	0.96	No

Two comparisons were statistically significant by Bonferonni criteria (p<0.008).

## Discussion

Handover refers to the transition of patient care from one provider to another. A “precarious exchange” has been a term applied to characterize the sometimes dangerously erroneous information contained within physician handover [11,25]. Since 2006, the Joint Commission clearly identified a lack of proper education around handover and handover curriculums [26]. Consequences of ineffective and incomplete handovers and the lack of dedicated handover curriculums and practices remain a major source of patient safety concern with deleterious consequences [26].

The CLER, published by the ACGME in 2016, made specific mention of the fact that many medical learning environments lack a dedicated curriculum to teach or evaluate effective communication between transitioning physicians and healthcare teams [19,22]. A number of handover curricula and tools have evolved over the years, the most prominent and well studied of which is the IPASS curriculum [1,3,7-9,12].

We introduced a handover bundle that not only introduced a formalized curriculum but also revised and remodeled the existing environment in which residents' hand over patient information at the change of shift. For our bundle, we utilized the SAFETIPS format due to the author’s comfort and familiarity with this model. Our bundle resulted in more statistically relevant resident communication with the maintenance of this improvement over time.

By providing residents with a standardized framework for handover, we encouraged the consistent and predictable transfer of both verbal and written communication, inclusive of essential patient information and key points without providing extraneous details. The curriculum encouraged residents to evaluate their patients via the SAFETIPS format both verbally and in written format in the EMR. Ongoing education and refresher sessions regarding the format helped to ensure that utilization would be more consistent and longitudinal in nature. Over a two-and-a-half-year period, our program experienced sustained improvement in resident communication, as it related to the handovers. As our data suggest, this improvement was noted not only by external evaluators on the handover evaluation tool but also internally by the residents using the UPDATED survey tool.

Our external evaluator survey tool focused on several key areas of physician communication: organization and efficiency, communications, content, clinical judgment, patient focus, and setting. All of these domains showed significantly improved scores as indicated by stacked dot plots in Figures 1-3. The data strongly supports the importance that all training programs have a structured handover communication curriculum in place. While an overall statistically significant improvement was noted from start to finish, a fair degree of variability existed between one post-implementation period to another. The lack of improvement seen from one post-curriculum implementation data point to the next, following the initial improvement, can be explained through a rapid post-curriculum implementation score improvement with a subsequent natural plateauing of scores associated with the skill acquisition and mastery in some of our categories. With intermittent refresher sessions, no significant erosion of scores was observed, suggesting the newly mastered skill set was maintained over time.

In the time period immediately following curriculum implementation, the handover survey category “clinical judgment” showed significant improvement, suggesting a greater clarity of communication regarding sick vs not sick patients, with a subsequent plateauing in scores (Tables 9, 10). Theoretically, once residents more clearly understood how to communicate which patients were of the highest acuity, this skill was maintained without significant change throughout the post-implementation periods. The handover category of “communication skills” afforded the opportunity to clarify expected responsibilities, encourage time for questions and clarifications, and eliminate irrelevant information while including key data points (Tables 5, 6). Once the skill set of clarification and opportunities for questions was presented, residents were able to successfully adopt and maintain this skill without erosion.

“Organization and efficiency” and “content” showed significant improvement in all comparative data points without a plateauing effect (Tables 3, 4, 7, 8, respectively). Presumably, efficiency and organization would continually improve and be further refined over time, as demonstrated by our data. Once provided a standard format for creating and prioritizing to-do lists and providing a rationale for the next steps in care (the “content” category of the handover evaluation survey), UPDATED evaluations indicated that residents were more confident making subsequent decisions on the next care shift and in addressing previously unanticipated needs. As this skill continued to improve over time and with refinement, a further statistically significant change was observed across and between most of the post-implementation periods. It may be worth considering that these areas of ongoing growth be further emphasized in subsequent refresher sessions with residents over time.

A review of existing curricula reveals that the categories of anticipatory guidance (in our survey category of “clinical judgment”) and to-do’s (in our category of “content”) as the most consistently present across handover curricula. Arguably, this information is among the most crucial data to be transferred from one team to another given the potential for a direct relationship to patient safety [27]. Future work may look to link improved communication scores with improved patient outcomes measures, a data point that has remained elusive in the medical literature given the number of confounding variables that encompass patient safety [4]. 

Handover survey categories of “patient focus” and “setting” showed statistical significance in improvement from pre- to post-implementation (Tables 11-14, respectively). However, in both categories, there was no significant improvement in scores between post-implementation time periods; these categories showed a plateau in improvement. It stands to reason that once an appropriate setting and parameters for handover location and conditions were established if adhered to, there would be little room for further improvement. Fortunately, this improvement was preserved, suggesting that improved habits were maintained, such as limiting possible internal team disruptions to handover content and location.

External handover interruptions remained relatively unchanged, despite the dedicated location and education taking place with other members of the patient care team (i.e., nursing). In a study by Habicht et al., their team correctly identified that handover interruptions remain prevalent, disrupt the handover communication process, and lend to erroneous communication which is well known to play a role in adverse patient outcomes [30]. Understanding the multifactorial etiology of interruptions and continuing to find effective ways to mitigate their occurrence remains an area of increased scrutiny in our work and others.

One interesting observation from our study was that teams were noted to more consistently start their handover process at the appropriately designated time once a formal curriculum was implemented (Table 1). However, sign out times became somewhat more variable when post-implementation periods were compared; the data were not included because it was only observational by the study team. While our curriculum included refresher courses on both the content of the oral and written components of the bundle, the physical space for handover was not always reviewed and revisited and observations did not happen at all post-implementation handover periods. The variability stresses the importance of institutional support of the initiative to ensure consistent, ongoing buy-in and the ongoing need for recurring educational updates with learners [1].

The trajectory of improvement as recorded by the trained observers followed a different pattern than that of the residents’ self-report. Resident scoring of handover (UPDATED) perceived the subsequent change post implementation differently. While overall there was a statistically significant improvement in the scores over time (Figure 3), this significance did not occur until comparing pre-implementation data with post-implementation period-2 and post-implementation period-3 data, corresponding with the nine- to 18-month post-implementation time period (Tables 19, 20). Scores compared between the pre-implementation and post-implementation period 1 were not statistically significant, nor were the post-implementation period 1 as compared to post-implementation period 2 or 3 statistically significant (Table 19 and 20). Altogether, this suggests a more gradual improvement in sign out as perceived by residents over a longer period of time. This may be related to the residents’ perceived need for ongoing practice and an expectation for a more gradual progression and adherence to a newly learned skill set.

When a handover evaluation Likert scale was completed by trained observers, there was a more significant improvement in sign out in the timeframe shortly after implementation with a plateauing effect over time. Resident evaluations (UPDATED) revealed no significant change in the initial post-implementation period, with a more significant change after a longer period of time, exposure, and practice. This is interesting and perhaps conflicts with existing data to suggest that physicians are inherently poor at objective self-assessments, usually erring toward over-competence and knowledge [28].

Limitations

A limitation of our study is the use of multiple students for observation of resident handover and resultant intra-observer variability. This was addressed by having the primary observer meet and train subsequent observers for the study. Onboarding observers attended several sessions with the primary observer to first evaluate residents independently but then discuss findings as a group to review ratings and discuss areas of variability (if any). Once all observers were found to give similar scores for observations, each observer was then allowed to evaluate handover independently. Completion of both the handover evaluation Likert scales and UPDATED tools are, inherently, subjective processes prone to variability; objective measures correlating the handover process to patient safety are challenging. 

As with our prior work, this study was again limited to one Pediatric residency training program within one Children’s Hospital. Institutional limitations prevented the dissemination of our tool to all training programs - an issue that prevents uniform dissemination and implementation. Although the use of standardized curricula is prevalent amongst programs, those curricula remain variable. Applicability to other care providers, such as Attending Physicians, should be another area of focus [29].

## Conclusions

Our study highlights the significance and importance of standardized handover curriculum - both verbal and written. It not only capitalizes on existing work but furthers the area of existing research by utilizing differing curricula to gauge applicability and generalizability. Implementation directly satisfies several of the CLER goals set forth by the ACGME. We have identified an evidence-based curriculum that improves resident communication and, with sustained education and support, those improvements are perpetuated over time.

Future goals

Expanding our findings outside of the inpatient, pediatric division to either other divisions or even departments is critical but challenging. Similarly, directly correlating physician communication with objective, patient safety data would lend continued credence to this work. An interesting area of study with limited research is how such training during residency carries over to the attending role. Similarly, for those physicians who lacked any standardized communication techniques during training, the idea of whether curriculum can be implemented and sustained at the Attending level remains to be seen.
